# A Randomized Clinical Trial of a Quitline Vaping Cessation Intervention: Baseline Characteristics of Young Adult Exclusive E-Cigarette Users Seeking Treatment

**DOI:** 10.3390/ijerph21060809

**Published:** 2024-06-20

**Authors:** Elizabeth G. Klein, Abigail B. Shoben, Kelly M. Carpenter, Kristina Mullis, Julianna M. Nemeth, Elizabeth Mayers, Katrina A. Vickerman

**Affiliations:** 1 College of Public Health, Ohio State University, 1841 Neil Avenue, Columbus, OH 43210, USA; shoben.1@osu.edu (A.B.S.); nemeth.37@osu.edu (J.M.N.); mayers.31@osu.edu (E.M.); 2 RVO Health, 1101 Red Ventures Drive, Fort Mill, SC 29707, USA; kcarpenter@rvohealth.com (K.M.C.); kmullis@rvohealth.com (K.M.); kvickerman@rvohealth.com (K.A.V.)

**Keywords:** vaping cessation, young adult, e-cigarettes

## Abstract

Despite interest in quitting vaping among young adults (YAs), little is known about characteristics of e-cigarette (EC) users seeking treatment. In this study, YAs aged 18–24 living in the United States interested in vaping cessation treatment were recruited to complete an online survey regarding demographics and EC use. Primary eligibility criteria were EC use on at least 20 days per month (no other tobacco use), and interest in quitting in the next month. We report descriptive statistics for those who did and did not complete a mandatory coaching call (*n* = 981). In this sample, most EC users reported high nicotine dependence, a history of unsuccessful quit attempts (including 29.4% with previous NRT use), along with stress, anxiety, and depression. There were few meaningful differences in demographics, EC use behaviors, or behavioral health factors between those who engaged with a phone coaching call (fully enrolled in study; *n* = 508), and those who did not (*n* = 473). YAs demonstrated interest in vaping cessation support, but there were no clear characteristics for the half who did not complete a coaching call. Vaping cessation program designers should consider tailoring for the self-reported behavioral health concerns present in this population.

## 1. Introduction

In the United States (US), current young adult (YA; 18–24 years of age) e-cigarette (EC) prevalence is estimated to range from 11.0% to 26.2%, with rising reports of daily EC use among YAs [1,2,3,4]. An estimated 39–57.5% of YAs made a quit attempt in the past year [5,6], and YAs expressed interest in cessation resources [7]. Despite active pursuit of cessation resources, few options tailored to YA EC users exist [8]. Historically, YA tobacco users, who were primarily smokers, had higher rates of spontaneous cessation attempts than adult tobacco users, but were less likely to use evidence-based methods [9]. Further, evidence is growing that barriers and facilitators for vaping cessation may be distinct from smoking cessation [8,10].

There is a growing need for research on effective cessation for EC users interested in quitting. Few fully-powered vaping cessation studies have been published. Graham et al. found a 24.1% 7-day abstinence rate at 7 months for YA EC users in an automated text messaging program, compared to 18.6% in the control condition [7], with exclusive EC users at baseline achieving higher rates of nicotine abstinence compared to those who co-used ECs and combustible cigarettes at baseline [11]. Qualitative research has suggested that despite the overlap in the reasons for quitting smoking and/or vaping, there are unique barriers and facilitators to vaping cessation among YA [10], resulting in a gap in the evidence for how best to engage YA EC users in effective cessation resources [8]. Despite heavy cell phone use and dependence [12], some data have suggested that the majority of YAs (90%) prefer not to talk on the phone [13], which has raised questions whether this age group would engage with cessation treatment that requires a one-on-one voice connection with a quitline coach.

The US 2020 Surgeon General’s Report on Smoking Cessation highlighted the need for effective, evidence-based vaping cessation interventions [14]. Specifically, effective interventions tailored to YA EC users are needed for successful and complete tobacco cessation among this priority population to disrupt addiction earlier in the life course. The present study describes a sample of treatment-seeking young adults (ages 18–24) who completed the baseline survey for a randomized clinical trial of a remotely delivered intervention to support EC cessation. The study required participants to complete at least one phone-based coaching call to enroll. Given the concerns about Yas’ willingness to talk on the phone, the objective of this paper is to describe the population of treatment-seeking YAs who exclusively vape and explore possible differences between those who engaged in phone-based coaching session(s) to those who did not. These findings will help characterize this population, about whom little is known, regarding use patterns, dependence, and behavioral health.

## 2. Materials and Methods

### 2.1. Overview

These data are from the Research and Innovation to Stop EC use among YAs (RISE) study, a randomized controlled trial testing a remotely delivered, vaping cessation intervention (ClinicalTrials.gov ID: NCT04974580). The RISE treatment was developed leveraging evidence-based tobacco cessation methods and protocols for smoking cessation: US phone-based quitline coaching, NRT, and digital cessation intervention [15]. In brief, all participants were offered 2 cessation coaching calls, the first call being required to fully enroll in the study, and then were randomized to one of 4 groups in the 2 × 2 design: nicotine replacement therapy (NRT) (8 weeks vs. none) and/or mHealth intervention (yes vs. no).

The present data are drawn from an online survey conducted to determine baseline characteristics of those who met the following eligibility criteria: (1) US-based individuals ages 18–24 years, (2) exclusive EC use [no other tobacco last 30 days; last 90 days if former smoker (100+ cigs/cigarillos in lifetime)], (3) EC use for 20+ days out of the last 30, (4) interested in quitting in the next 30 days, (5) smartphone user, (6) eligible for use of nicotine replacement therapy (NRT; based on 5 use exclusions specific to cardiovascular conditions as a contraindication), (7) not pregnant or breastfeeding, and (8) the ability to read and write in English. Funded as part of the End Nicotine Addiction in Children and Teens (ENACT), the RISE study focused on younger EC users to support cessation efforts [16]. The current study focused on exclusive EC users due to the lack of literature on NRT for exclusive vaping and subsequent need for research [17]. Without sufficient additional content addressing the relative risk of tobacco products, there is a potential risk that focusing on the importance of vaping cessation could have the iatrogenic effect of increasing cigarette use in dual users.

### 2.2. Procedures

National participant recruitment was conducted primarily via a recruitment firm (https://climb.care/) where advertisements were placed on Instagram and Facebook; additional methods included other social media or online methods (Reddit, StudySearch, etc.). A two-step recruitment process was conducted, starting with an initial screening survey hosted by the recruitment firm. After initial screening for eligibility by the firm, those who met all eligibility criteria listed above were referred to an online survey through a web-based survey platform (Qualtrics, Seattle, WA, USA) where individuals provided a digital signature for written study consent prior to completing a baseline survey. Individuals who completed the baseline survey and were eligible for the study were randomized to receive NRT (yes vs. no) and/or the mHealth program (yes vs. no; text message program with links to videos, podcasts, and other online learning content), delivered using a factorial design. Quality assurance reviews of eligible participants were conducted by research staff to evaluate participant responses for inconsistencies, fraud, or other concerns in the baseline survey responses prior to treatment enrollment; methods included valid address verification using the USPS zip code lookup tool and duplicate information checks.

As a final enrollment step, an attempt to contact each participant was made by a trained quitline coach associated with the Quit For Life tobacco cessation program via RVO Health (formerly Optum Health—https://rvohealth.com/). Quitline program protocols have been shown to be effective and cost effective in previous studies and evaluations [18,19,20,21]. Quitline coaches had previously received 240 h of tobacco training plus ongoing supervision, and received 4 h of training specific to the RISE study with ongoing feedback. Individuals who did not complete the first coaching call after 5 attempts were not fully enrolled in the study and did not receive study interventions. Participants who completed at least one coaching call were fully enrolled in the study and received a second proactive call around their planned quit date. Recruitment was conducted over 15 months (July 2021 through September 2022). Participants received a USD 40 e-gift card following the baseline survey participation and completion of the cessation coaching call.

### 2.3. Measures

Baseline survey measures included the following demographic factors: age, gender identity, race/ethnicity, educational attainment, chronic conditions, employment status, using validated measures from the PhenX toolkit on tobacco use as well as the Population Assessment of Tobacco and Health [22,23]. Tobacco use behaviors included types of ECs, frequency of use, reasons for use, nicotine content, EC use at home/work, knowledge and beliefs about ECs, cessation interests, and dependence as measured by the PROMIS-E and Penn State E-cigarette Dependence scales [22,24,25]. Behavioral health metrices included the PHQ-2 [26], GAD-2 [27], and PSS-4 [28] for depression, anxiety, and stress, respectively.

### 2.4. Analysis

In the present analysis, all eligible individuals who provided consent and completed the baseline survey were included (*n* = 981). Descriptive statistics (means and standard deviations or percentages) were used to characterize the entire eligible sample. Those individuals who completed the first call with a quit coach (thus, enrolled in the RISE study; *n* = 508) were compared to those who met eligibility criteria but did not complete the coaching call (*n* = 473). Formal statistical testing for differences between these groups was not conducted as the goal was to descriptively understand whether potentially meaningful differences exist, rather than strict statistical significance. Further, as this study is an analysis of data collected for the clinical trial, it was not powered or designed to provide a confirmatory answer to questions of differences between these two groups. This manuscript summarizes data from the RISE baseline cross-sectional, online survey. In the following section, the results are summarized with descriptive statistics and do not include statistical comparisons since these analyses were not related to hypotheses in the RISE trial, so descriptive results are used to avoid misinterpretation [29]. Data were analyzed using Stata (StataCorp, College Station, TX, USA).

## 3. Results

**Figure 1:**
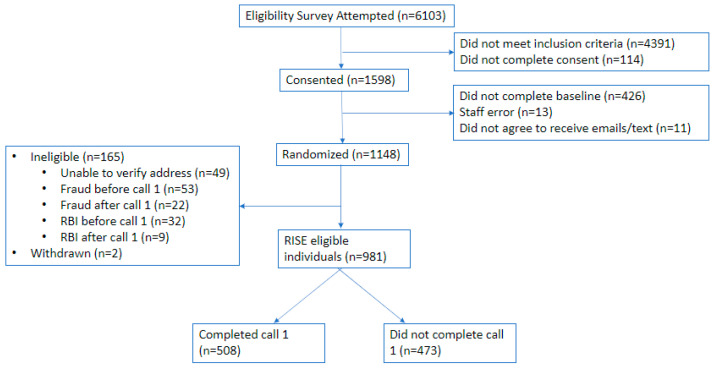
CONSORT diagram for RISE study. RBI: randomized but ineligible.

Over the 15-month study period, the eligibility survey for the RISE study was started 6103 times; 72% (*n* = 4391) did not meet study eligibility criteria, with the majority due to current other tobacco use (61%; *n* = 2701/4391) (Figure 1). Following eligibility, 1598 individuals consented to participate. Among those who consented to participate, 426 did not complete all required survey elements and 24 were removed due to error or additional eligibility review. The resulting 1148 individuals were randomized to one of four RISE study conditions. Following the quality assurance review by research staff, an additional 134 individuals were deemed ineligible (most due to fraud or invalid address), 2 withdrew from the study, and 473 did not complete at least one coaching call (referred to here as “non-participants”); the remaining individuals who completed the coaching call (*n* = 508) were considered RISE study participants (referred to here as “participants”).

### 3.1. Demographic Comparisons

The majority of the total sample of YA EC users (*n* = 981; participants and non-participants) were aged 18–21 (54.7%) and female (72.6%); 3.8% of the sample identified as nonbinary gender (Table 1). Most of the sample reported Caucasian (70.0%) race, 9.0% Black or African, 8.5% Hispanic or Latinx ethnicity, and 12.5% reported more than one race/ethnicity. Most of the sample reported some college or vocational training (57.4%), while 26.5% reported being a college graduate and 16.1% reported being at or below high school graduation. Percentages of these demographics were quite similar between participants and non-participants, with the largest difference that participants were slightly younger (57.1% aged 18–21) than non-participants (52.2%).

For tobacco use behaviors (Table 2), the majority of values were similar between participants and non-participants. The majority of the sample used ECs daily (77.7%) and had been using them for at least one year (86.9%). Nearly all (95.8%) reported using a flavored EC, and most used disposable devices (57.5%) overall. A slightly higher percentage of participants reported disposable EC use (61% vs. 53.9%) and a lower percentage reported buying pre-filled cartridges (22.5% vs. 28.6%), compared to non-participants. Using the Penn State E-cigarette Dependence Index [30], most participants were categorized as heavily dependent (70.9%); however, 21.2% had missing data on one or more items, thus the full-scale score could not be calculated for these individuals. The four-item PROMIS-E assessment of EC dependence had missing data for 2.1% of the sample; the mean t-score was 58.6, which is nearly one standard deviation above the mean or approaching moderate severity of dependence symptoms [31]. Using a 10-point scale where 10 equals highly motivated to quit, the sample scored 7.8 (SD = 1.7) overall. The reasons reported for wanting to quit vaping were desiring freedom from addiction (88.8%), health concerns (87.6%), cost (73.4%), the impressions of others (35.9%), or other reasons (7.4%). Nearly all individuals had previously attempted to quit vaping (87.4%), with most attempting three or more times (64.3%). Most reported a longest EC abstinence period of 1–6 days (29.7%) or 7–30 days (19.3%) with 18.2% reporting previous abstinence of 3 months or more. Only a third previously used a cessation medication to quit vaping (31.5%), primarily NRT in the form of patch, gum, or lozenge (29.3% ever use). Current use of any type of NRT at the time of the baseline survey was 7.5%; non-participants reported current NRT use at a higher rate (9.7%), compared to participants (5.5%).

### 3.2. Behavioral Health Comparisons

Behavioral health concerns were high in the total sample (Table 3). Nearly half (44.8%) screened positive for depression (PHQ-2), more than half (55.4%) screened positive for anxiety (GAD-2), and most (73.1%) screened positive for high stress (PSS-4). Four in five (88.8%) reported alcohol use in the past month, and more than half the sample (57.4%) reported binge drinking at least once a month, with 1–2 days per month the most common. The majority reported using cannabis (59.7%) in the past month. These values were largely similar between participants and non-participants, with non-participants with a higher proportion reporting anxiety (59% vs. 52%) and depression (47% vs. 43%).

## 4. Discussion

The high demand for cessation support among YA EC users experienced during recruitment for this randomized clinical trial is consistent with the Truth Initiative’s rapid enrollment of youth and young adults into their text-based EC cessation intervention [7]. Tobacco control researchers and practitioners should be attentive to this expressed interest in intervention that will support disruption of nicotine addiction early in the life course [32].

Surprisingly, our analyses revealed few meaningful differences among YAs who did and did not engage with the phone coaching call. Despite our examination across demographic, tobacco specific, and behavioral factors, we only identified several small differences between those YAs who completed the first coaching call and ultimately enrolled in the study compared to those who did not. Specifically, participants who completed the first call reported being age 18–21 and using disposable ECs at slightly higher rates and reported current use of a cessation aid, using pre-filled cartridge ECs, and meeting cut-offs for depression and anxiety screening at slightly lower rates (all differences: 4–7 percentage points). While individuals with higher social anxiety report less interest in making voice calls by phone [33], we did not see large differences in our sample between those who opted out of the quitline call compared to those who did. Due to the provision of NRT as part of the study intervention and the lack of previous trials examining NRT for vaping, the RISE study team elected to require a coaching call as part of the study enrollment process so that YAs would speak with an expert quit coach for NRT dosing and education. While we expect that YAs may be less interested in speaking on the phone compared to older adult populations, some YAs make calls to state quitlines in the U.S [34]. State quitline infrastructure was used to implement the RISE intervention because quitlines are designed to reach and serve large populations with evidence-based, low-barrier, cost-effective treatment. Digital-only interventions have shown mixed results for some YA tobacco users, mostly smokers, to successfully quit [35]; while data are limited, engagement with text and/or digital interventions among YA EC users has shown promise [7,8]. Still, as with other healthcare interventions, one-on-one interaction with an expert may be needed, potentially through a menu of services or stepped care approach [36]. Future studies should examine intervention methods that do not require phone engagement for participation, perhaps by using more novel methods of promoting coach interactions such as chat or live texting [37].

The overwhelming cause of RISE study ineligibility was dual use of ECs with combustible tobacco products. Given the strong evidence of the substantial health risks of combustible tobacco use [38], the development of effective cessation strategies for dual users are needed. Graham et al. [7] found that the This is Quitting text-based vaping cessation intervention was effective for both exclusive EC users and dual users of ECs and cigarettes, but it was less effective for dual users. Research to support effective vaping cessation while also addressing dual use and the paramount importance of cigarette abstinence is needed to provide better support to YAs seeking cessation support [17].

Desiring freedom from addiction, health concerns, and cost were highly endorsed as motivators for vaping cessation. These findings are consistent with work that Amato and colleagues reported on the subjective experiences motivating quit attempts and treatment-seeking for EC users, where half (50.9%) reported interest in quitting for health improvement [39]. Importantly, the RISE study sample was highly nicotine dependent, nearly a full standard deviation (10 points) above the reference mean of adults who vape [31]; this result may be a byproduct of recruitment of frequent EC users—the population for whom NRT would most likely be indicated. Additionally, high rates of co-use of alcohol, cannabis, anxiety, and stress were present in our population. High rates of EC, tobacco, and cannabis co-use in younger populations have also been highlighted in other samples [40,41], with calls for more holistically addressing substance use among youth and YAs. Considering the overall health and wellbeing of this YA population, consideration must be given in intervention development to support individuals using ECs to cope with distress, and to avoid increasing rates of cannabis or risky alcohol use when quitting nicotine [42].

The present study has important limitations to consider. While our study sample was drawn from the entire US., it was not designed to be nationally representative. Relatedly, our study recruitment used advertisements on popular social media platforms, which limited our population to YAs who engage in those specific sites (Instagram, Facebook). Additionally, the present study focused on exclusive, regular (20 or more days out of the last 30 days) EC users, due to one of the study aims to test NRT for exclusive vaping cessation. The remote-delivery of all RISE study contacts allowed for conducting this trial during the latter phase of the COVID-19 pandemic. This unique timeframe may have impacted participant characteristics and behaviors, including stress levels, and the generalizability of this sample; for example, EC users have reported that the pandemic impacted their EC behaviors, generally increasing use [43]. All of these elements impact the population to which these findings may generalize. In addition, our online recruitment methods and fully remote design introduced challenges with fraud, also seen by other tobacco researchers [44]. We assume there is a small percentage of the 473 “non-participants” who did not complete the first coaching call who may have been fraudulent participants. In future studies, we plan to improve fraud checks at the time of consent. Finally, although the present study captured a range of demographic, psychosocial, tobacco use, and behavioral factors, there may be unknown external factors that contributed to differences in those who completed quit coach calls; future examinations for cessation intervention preferences and exploration of cognitive barriers [45] may help to explicate the reasons YAs may or may not be willing to engage in one-on-one coaching with a nicotine cessation expert by phone, and inform motivation messages for individuals who may benefit most from engaging in one on one coaching [43].

## 5. Conclusions

There is a great deal of interest and demand for vaping cessation among exclusive EC users and dual users in the US Tobacco control researchers and practitioners should leverage this interest to support disruption of nicotine addiction early in the life course. Future research is needed to understand and tailor to the preferences for cessation intervention modes of delivery, how best to engage YAs seeking treatment, and whether tailoring for increased rates of stress and behavioral health concerns in this population improves treatment.

## Figures and Tables

**Table 1:** Young adults in the RISE study: Comparison of participants and non-participants.

Demographics	Participants (*n* = 508)	Non-Participants (*n* = 473)	Total (*n* = 981)
Age			
18–21	57.1% (290)	52.2% (247)	54.7% (537)
22–24	42.9% (218)	47.8% (226)	45.3% (444)
Gender			
Female	71.3% (362)	74.0% (350)	72.6% (712)
Male	24.8% (126)	22.4% (106)	23.7% (232)
Transgender female	0.2% (1)	0.6% (3)	0.4% (4)
Transgender male	2.0% (10)	1.5% (7)	1.7% (17)
Other Identity	1.8% (9)	1.5% (7)	1.6% (16)
Race			
Caucasian (only)	69.0% (350)	71.1% (334)	70.0% (684)
Black/African (only)	8.5% (43)	9.6% (45)	9.0% (88)
Hispanic/Latinx (only)	9.5% (48)	7.5% (35)	8.5% (83)
All others	13.0% (66)	11.9% (56)	12.5% (122)
Education			
≤High school graduate	15.1% (76)	17.2% (81)	16.1% (157)
Some college/vocational training	59.3% (299)	55.3% (260)	57.4% (559)
≥College graduate	25.6% (129)	27.5% (129)	26.5% (258)

**Table 2:** Young adults in the RISE study: tobacco use behaviors of participants and non-participants.

	Participants (*n* = 508)	Non-Participants (*n* = 473)	Total (*n* = 981)
EC use			
30 out of 30 days	77.9% (395)	77.4% (366)	77.7% (761)
<every day	22.1% (112)	22.6% (107)	22.4% (219)
EC use duration			
12 months or more	86.4% (439)	87.3% (413)	86.9% (852)
6–12 months	6.9% (35)	6.6% (31)	6.7% (66)
<6 months	6.7% (34)	6.1% (29)	6.4% (63)
Type of EC device			
Disposable	61.0% (303)	53.9% (252)	57.5% (555)
Buy pre-filled cartridges	22.5% (112)	28.6% (134)	25.5% (246)
Refill independently	16.5% (82)	17.5% (82)	17.0% (164)
Penn State Dependence scale			
Missing	19.7% (100)	22.8% (108)	21.2% (208)
0–3 (not dependent)	0 (0%)	0 (0%)	0 (0%)
4–8 (low)	5.9% (24)	7.7% (28)	6.7% (52)
9–12 (medium)	22.3% (91)	22.5% (82)	22.4% (173)
13+ (high)	71.8% (293)	69.9% (255)	70.95 (548)
PROMIS-E			
T-scores	58.6 (8.3)	58.6 (8.6)	58.6 (8.5)
Missing	2.2% (11)	2.1% (10)	2.1% (21)
Motivation to quit scale (with 10 rated as highly likely)	7.8 (1.7)	7.7 (1.8)	7.8 (1.7)
Reasons for quitting			
Freedom from addiction	91.1% (463)	86.3% (408)	88.8% (871)
Health concerns	87.8% (446)	87.3% (413)	87.6% (859)
Cost	74.2% (377)	72.5% (343)	73.4% (720)
Others’ impression	36.6% (186)	35.1% (166)	35.9% (352)
Other reasons	7.8% (40)	7.0% (33)	7.4% (73)
Ever try to quit vaping	88.1% (446)	86.5% (405)	87.4% (851)
Previous quit attempts			
1–2	34.2% (150)	37.4% (150)	35.7% (300)
3–4	41.5% (182)	42.6% (171)	42.0% (353)
5+	24.4% (107)	20.0% (80)	22.3% (187)
Longest EC abstinence			
<24 h	10.2% (52)	12.7% (60)	11.4% (112)
1–6 days	35.1% (178)	39.5% (186)	37.2% (364)
7–30 days	20.7% (105)	17.8% (84)	19.3% (189)
1–3 months	16.5% (84)	11.2% (53)	14.0% (137)
>3 months	17.5% (89)	18.9% (89)	18.2% (178)
Ever use of any aid to quit vaping	30.5% (155)	32.6% (154)	31.5% (309)
Ever use of nicotine replacement therapy (NRT) to quit vaping	28.2% (143)	30.4% (144)	29.3% (287)
Current cessation aid use			
Any cessation aid	5.5% (28)	9.7% (46)	7.5% (74)
Patch	1.2% (6)	3.2% (15)	2.1% (21)
Gum	2.2% (11)	4.4% (21)	3.3% (32)
Lozenge	2.0% (10)	2.3% (11)	2.1% (21)
Any NRT (P/G/L)	3.7% (19)	6.8% (32)	5.2% (51)

**Table 3:** Young adults in the RISE study: behavioral health and other substance use among participants and non-participants.

	Participants (*n* = 508)	Non-Participants (*n* = 473)	Total (*n* = 981)
PHQ-2 ≥ 3 (depression)	42.9% (218)	46.7% (221)	44.8% (439)
GAD-2 ≥ 3 (anxiety)	52.0% (264)	59.0% (279)	55.4% (543)
PSS-4 ≥ 6 (stress)	73.0% (371)	73.2% (346)	73.1% (717)
Any alcohol use in the past month			
None (0 days)	18.5% (93)	20.1% (94)	19.2% (187)
1–2 days	25.0% (126)	18.6% (87)	21.9% (213)
3–5 days	21.2% (107)	24.8% (116)	22.9% (223)
6–9 days	17.9% (90)	18.2% (85)	18.0% (175)
10+ days	17.5% (88)	18.4% (86)	17.9% (174)
Frequency of binge drinking in the past month			
None (0 days)	43.7% (221)	41.1% (193)	42.4% (414)
1–2 days	29.3% (148)	27.5% (129)	28.4% (277)
3–5 days	14.2% (72)	17.2% (81)	15.7% (153)
6–9 days	7.9% (40)	8.5% (40)	8.2% (80)

## Data Availability

The original data presented in the study are openly available at https://github.com/klein232/RISE.git.
